# Vomiting of Fluids, but Not of Solids

**Published:** 1895-12

**Authors:** A. H. Tompkins

**Affiliations:** Jamaica Plain, Mass.


### VOMITING OF FLUIDS, BUT NOT OF SOLIDS.

Jamaica Plain, Mass., October 29th, 1895.

Editor of The Homœopathic Physician :

In the course of your article on Ars-alb., in October Homœopathic Physician, you give Bapt. as having " vomiting of fluids but not of solids." As I have always connected that symptom with Bismuth and never with Bapt., I thought it might have been a slip of the pen with you. I have verified it as regards Bismuth. Very truly yours,

A. H. Tompkins.

[Dr. Tompkins is, most likely, right in this matter. A more careful examination of the notes from which the symptom was taken shows that it was not given by Dr. Adolph Lippe at all, but came to the Editor through conversation with Dr. Lippe's son, Constantine. The Editor may have misunderstood what

Dr. Constantine Lippe said, for while an inspection of several books of materia medica does not show the symptom as stated under either remedy, yet as the symptoms are given in the books the testimony is in favor of Bismuth.—EDITOR.]

JAMAICA PLAIN, Mass., Nov. 2d, 1895.

EDITOR OF THE HOMŒOPATHIC PHYSICIAN:

Your favor of October 31st is received. In regard to the Bismuth symptoms of *vomiting fluids and retaining solids*, I have been as unsuccessful as yourself in finding it in any materia medica. The nearest to it is the "vomiting all fluids," in Hering's *Condensed*, third edition, p. 199, as this implies that fluids are differently treated by the stomach than solids. However, I remember having been given this by my professor of materia medica twenty years ago, and I am sure I have come upon it in my reading of homœopathic literature many times since. Yes, I find it in Bell's *Therapeutics of Diarrhœa*, third edition, p. 34: "Vomits water only; food is retained." Liquid food could hardly be retained, of course, if taken at the same time with the water.

I remember a very difficult case of colic and vomiting in an infant, which Bismuth enabled me to cure, and in which the vomiting of the whey with retention of the curds of the milk caused me to study and finally prescribe this remedy.

Having found so much in Dr. Bell's book, I turned to see what it said about Bapt., but found nothing to hint at special intolerance of water or fluids. On the contrary, the discrimination, such as it is, seems to be in favor of fluids under Bapt. Bell says (and Hering's *Condensed* has it in nearly the same words): "Child can take nothing but liquids; the slightest amount of solid food causes gagging."

So, it would seem that clinical experience, at least, gives Bismuth a right to be used when the stomach rejects fluids and retains solids, if other symptoms conspire to indicate it, while Baptisia has no voucher hitherto in our literature for such a characteristic. Very truly yours,

A. H. TOMPKINS.

